# Impact of WeChat guidance on bowel preparation for colonoscopy: a quasi-experiment study

**DOI:** 10.1038/s41598-023-37435-z

**Published:** 2023-07-18

**Authors:** Yifang Guan, Yanjun Song, Xiaona Li, Aijun Zhang, Ruyuan Li

**Affiliations:** Department of Gastroenterology, Qilu Hospital (Qingdao), Cheeloo College of Medicine, Shandong University, Qingdao, China

**Keywords:** Diseases, Gastroenterology

## Abstract

Colonoscopy is a standard procedure for screening, monitoring, and treating colorectal lesions. To explore the impact of WeChat guidance on bowel preparation before colonoscopy. This quasi-experiment study included patients who underwent colonoscopy at Qingdao Endoscopy Center between March 2016 and September 2016. The primary outcome was bowel preparation quality (Ottawa score), the secondary outcomes were intubation time, withdrawal time, adenoma detection rate (ADR), and adverse reactions. Finally, 588 patients were included and divided into the WeChat guide (n = 295) and the non-WeChat guide (n = 293) groups, they were comparable in baseline characteristics. The Ottawa score (1.59 ± 1.07 vs. 6.62 ± 3.07, P < 0.001), intubation time (6.47 ± 1.81 vs. 11.61 ± 3.34, P < 0.001), withdrawal time (13.15 ± 3.93 vs. 14.99 ± 6.77, P < 0.001), and occurrence rate of adverse reactions (2.0% vs. 5.5%, P = 0.029) were significantly lower in the WeChat guide group than those in the non-WeChat guide group. ADR was significantly higher in the WeChat guide than that in the non-WeChat guide group (1.47 ± 2.30 vs. 0.84 ± 1.66, P < 0.001). WeChat guidance might improve the quality of bowel preparation and adenoma detection rate, shorten the time of colonoscopy, and reduce adverse reactions in bowel preparation.

## Introduction

Colonoscopy is a standard procedure for screening, monitoring, and treating colorectal lesions. Thorough intestinal preparation is required before undergoing a colonoscopy. It makes colonoscopy easier to conduct, decreases intubation time, saves medical expenditures for the patient, and enhances colonoscopy diagnostic accuracy and treatment safety^1,2^. Currently, various bowel preparation drugs and methods are available to improve the effectiveness of bowel preparation; however, approximately 33% of patients still exhibit poor bowel cleansing in clinical practice^3^. This reduces the lesion detection rate and delays the diagnosis and treatment of intestinal diseases^4,5^.

As bowel preparation requires dietary modification, laxative consumption, and adherence to strict timelines and guidelines, patient cooperation is critical in a successful colonoscopy. Traditionally, patients are guided on bowel preparation using written or verbal instructions. Recently, numerous studies have explored technological interventions to improve patients’ education and cooperation in bowel preparation^6^. For example, a couple of studies have sought digital tools to enhance patients’ compliance with bowel preparation instructions and improve the quality of bowel cleansing^7–9^. Walter et al.^7^ used a smartphone application to reinforce patient education and adherence to bowel preparation guidelines and showed that the patients who used the app had greater compliance, better bowel cleanliness, and a more successful colonoscopy^7^. Still, the role of social media apps on bowel preparation guidance remains unclear.

WeChat is an intelligent Chinese instant messaging and social media app that supports voice messaging, video calls, pictures, and text and provides various interactive functions such as public platforms, moments, and push notifications. This study aimed to explore the impact of WeChat guidance on bowel preparation before colonoscopy.

## Methods

### Study design and patients

This quasi-experiment study included patients who underwent colonoscopy at Qingdao Endoscopy Center, Qilu Hospital, Shandong University between March 2016 and September 2016 using a convenience sampling method. Inclusion criteria were as follows: (1) had appointment for colonoscopy for gastrointestinal symptoms (such as abdominal pain, diarrhea, constipation, and hematochezia), family history of colorectal cancer or cancer screening. (2) aged between 18 and 70 years. Exclusion criteria were as follows: (1) history of previous colonoscopy or colorectal surgery; (2) suspected gastrointestinal obstruction and perforation, dysphagia, gastroparesis, severe underlying cardiopulmonary disease, severe hematological disease, hemodynamic instability, cerebrovascular accidents within the last three months, terminal-stage chronic renal failure, acute or sub-acute hypertension, diabetes, pregnant and lactating women, or mental abnormalities leading to noncompliance with the examination. Patients were allocated to Wechat guide group (appointment order was uneven number) and non-WeChat guide group (appointment order was even number) according to the appointment order. This study was approved by the Ethics Committee of Qilu Hospital of Shandong University [No: KYLL-2020043]. Written informed consent was obtained from all patients.All methods were performed in accordance with the guidelines ESGE and regulations of our center.

### Intervention

#### Drugs for bowel preparation

Patients in both groups were administered 137.15 g (1 sachet) polyethylene glycol electrolytes powder (II) (He Shuang) (Wanhe Pharmaceutical, Shenzhen, China; SFDA approval No. H20030828) for bowel preparation. The bowel preparation solution was prepared 4–6 h prior to colonoscopy^10^ by dissolving one sachet of He Shuang in 2000 mL of cool boiled water. The patients were administered 250 mL of the solution every 10 min to ensure completion within 2 h.

#### Instructions for bowel preparation

All patients received routine bowel preparation instructions at the time of registration. They were advised to abstain from food with high fiber content, dark foods and foods containing seeds four days before the examination. On the other hand, easy-to-digest foods were recommended. Patients with constipation or bedridden for a long time were administered a laxative or a gastric motility agent 2–3 days in advance. A day before the examination, patients were advised to drink plenty of water and eat a liquid or semi-liquid diet. On the day of the colonoscopy, patients were told to fast. Then, they were informed of the importance of bowel preparation, possible side effects, and how to manage them. Finally, a leaflet with detailed instructions on bowel preparation was distributed to patients.

Patients in the WeChat guide group were invited to join a WeChat Group by scanning the WeChat QR code at their appointment time, and were given additional WeChat guidance. They accessed to WeChat independently or through family members living with them. The WeChat instructions were as follows: (1) The importance of bowel preparation, including improving the rate of early adenoma and cancer detection and reducing pain. (2) Dietary requirements: Pictures of recommended foods and forbidden foods were sent to patients, and were informed of the feasible combinations of daily recipes before the examination. (3) Patients with constipation or bedridden for a long time were reminded to administered gastric motility agents 2–3 days in advance. (4) Medication administration instructions: Patients were informed in detail of the medication administration process with flow charts and video simulations. (5) Adverse reaction response: Patients were informed of the possible adverse conditions during medication administration and the response measures. In the event of any other adverse reactions not listed or other questions about the colonoscopy, patients were encouraged to contact the endoscopy center via WeChat at any time. The endoscopy nurse sent instructions based on the examination time appointed by the patients. Specifically, items (1), (2), and (3) were sent 3–4 days before the examination, items (2), (3), and (4) were sent 2 days before the examination, and items (2), (3), (4), and (5) were sent a day before the examination. Patients were reminded to check the WeChat content between 9–10 am and 16–17 pm daily. After defecation, the pictures of different degrees of defecation statuspatients were sent to the patients to guide them to self-evaluate their defecation preparation.

#### Apparatus and operator selection

All patients were examined by five senior endoscopists using a PENTAX EPK i50 colonoscope. All colonoscopies were performed between 13:30 and 16:30 pm.

### Outcomes

The primary outcome was bowel preparation quality, the secondary outcomes were intubation time, withdrawal time, adverse reactions, and adenoma detection rate.

Experienced endoscopic surgeon and nurse who were blinded to the group allocation assessed the bowel preparation quality of each patient using the Ottawa bowel preparation scoring system^11^ (total score of 14), according to the cleanliness of the three parts of the colon: cecum and ascending colon, transverse and descending colon, and rectum and sigmoid colon, as well as the amount of fluid residue in the whole bowel. The cleanliness of the bowel was scored on a five-point scale as follows: 0 (satisfactory), no fluid or solid residue; 1 (good), a small amount of fluid or trace solid residue; 2 (acceptable), a moderate amount of fluid, not affecting observation; 3 (poor), a large amount of fluid and solid residue, affecting observation and requiring pumping; 4 (extremely poor), a large amount of solid fecal residue, not observable. The liquid residue was divided into three classes: 0, small amount; 1, medium amount; 2, large amount.

One researcher collected information during the bowel preparation process. The researcher did not participate in developing and delivering the colonoscopy and bowel preparation protocols for patients by Wechat. The study collected age, gender, appointment to examination time, adverse reactions during bowel preparation, Ottawa score, intubation time, withdrawal time, and adenoma detection rate.

### Statistical analysis

IBM SPSS Statistics, version 22.0 (IBM Corp., Armonk, N.Y., USA) was used for statistical analysis. Continuous data were expressed as mean ± standard deviation (SD) and compared by *t* test. Categorical data were expressed as n (%), and compared by the Chi-square test. A two-sided P < 0.05 was considered statistically significant.

### Ethics declarations

This study was approved by the Ethics Committee of Qilu Hospital of Shandong University [No: KYLL-2020043].

### Patient consent

Written informed consent was obtained from all patients.

## Results

A total of 660 patients were screened, and 72 patients were excluded due to pain or hypersensitivity (preventing them from adhering to the protocol), difficulty passing the scope due to stenosis from colon cancer, and abnormal vital signs prompting the termination of the examination. Finally, 295 and 293 patients were included in the WeChat and non-WeChat guide groups, respectively. They were comparable in age, sex, appointment to examination time, and history of constipation between groups (Table 1). No significant differences in baseline characteristics such as the number of cases, age, sex, appointment to examination time, and history of constipation were found between the two groups of patients (Table 1).

**Table 1:** Comparison of the baseline characteristics of patients in the WeChat and non-WeChat guide groups.

Characteristics	WeChat guide group (n = 295)	Non-WeChat guide group (n = 293)	P
Age (years)	54.00 ± 11.76	55.62 ± 11.44	0.137
Sex			0.904
Male	161	158	
Female	134	135	
Appointment to examination time (days)	3.64 ± 0.903	3.69 ± 0.837	2.417
History of constipation	27	24	0.679

Data were expressed as mean ± SD or n.

The Ottawa score (1.59 ± 1.07 vs. 6.62 ± 3.07, P < 0.001) was significantly lower in the WeChat than that in the non-WeChat guide group. In addition, the intubation time and withdrawal time were significantly shorter in the WeChat guide group (6.47 ± 1.81 and 13.15 ± 3.93, respectively) than those in the non-WeChat guide group (11.61 ± 3.34 and 14.99 ± 6.77, respectively) (both P = 0.001). Additionally, the adenoma detection rate was significantly higher in the WeChat guide group than that in the non-WeChat guide group (P = 0.001). Similarly, the occurrence rate of adverse reactions was significantly lower in the WeChat than that in the non-WeChat guide group (2.5% vs. 5.5%, respectively, P = 0.029) (Table 2).

**Table 2:** Comparison of intubation time, withdrawal time, adverse reactions, and adenoma detection results between the two groups.

Characteristics	WeChat guide group (n = 293)	Non-WeChat guide group (n = 295)	P
Ottawa score	1.59 ± 1.07	6.62 ± 3.07	0.001
Intubation time (min)	6.47 ± 1.81	11.61 ± 3.34	0.001
Withdraw time (min)	13.15 ± 3.93	14.99 ± 6.77	0.001
Number of adenoma detected	1.47 ± 2.30	0.84 ± 1.66	0.001
Adverse reactions	6 (2.5%)	16 (5.5%)	0.029

Data were express as mean ± SD or n.

## Discussion

This study shows a significantly lower Ottawa score, shorter intubation and withdrawal times, greater adenoma detection rate and fewer adverse reactions in patients that received bowel preparation instructions via WeChat than those who used conventional verbal and written instructions, suggesting that WeChat guidance might improve the quality of bowel preparation.

New services, including medical and social security services that rely on the internet as a carrier, are developing rapidly in China. With the dramatic evolution of social media, instant messaging services such as WeChat have gradually replaced traditional social communication media such as messaging and multimedia messaging services or online education. WeChat has the advantages of easy and fast information dissemination, broad reach, high interactivity, timely communication, and low cost^12^. Thus, it can provide a platform for easy communication and interaction between endoscopy nurses and patients. Illustrated bowel preparation instruction content can be sent to patients via WeChat. Even if some patients cannot or do not know how to use WeChat, they can access it with the help of their close family members.

The lower Ottawa score in the WeChat guide group aligns with previous studies that used smartphone applications to provide bowel preparation instruction^13–15^. The mean Ottawa score in the WeChat guide group was much smaller than that in the non-WeChat guide group. Similarly, Kang et al.^14^ compared patients that received WeChat instructions with those who only received conventional guidance and demonstrated that the former had a significantly lower Ottawa score (3.6 ± 1.7 vs 4.5 ± 1.8). Furthermore, a meta-analysis of six studies concluded that patients guided with smartphone apps have better bowel cleansing and improved colonoscopy examination outcomes compared to those who used conventional preparation instructions^16^. This study further illustrates shorter colonoscopy sessions in the WeChat guide group: both intubation and withdrawal times were shorter, indicating a more qualitative bowel cleansing. The non-WeChat guide group reported approximately twice as many adverse reactions as the WeChat guide group. Adverse events are more prevalent when the bowel is inadequately prepared^17,18^. By making patients well informed and more compliant, smartphone-based instructions improve bowel preparation quality and thus reduce the adverse effects^9^.

Detection and subsequent resection of adenoma reduce the morbidity and mortality of colon cancer^19^. However, Menees et al. reported that poor bowel preparation could lead to a 22 to 48% misdiagnosis rate for colorectal lesions^20^. Adequate bowel preparation, a prerequisite for high-quality colonoscopy, can improve the detection rate of adenomatous polyps^21^. In this study, the patients in WeChat guidance group had significantly higher adenoma detection rates. Similar findings have been previously reported^7,9,15^. Bowel preparation is a complex process involving dietary control, the administration of many laxatives, and appropriate psychological preparation^22^. A variety of factors can affect bowel preparation, including the type of bowel cleanser used, method of administration, time interval between bowel cleanser administrations, appointment to examination interval, and the baseline patient's status like sex, age, concurrent disease, and compliance^11,23^. However, the scarcity of medical resources inevitably prolongs the interval between colonoscopy appointments and subsequent examinations. Some authors showed that the delay between the appointment date and the colonoscopy is significant in the poor quality of bowel preparation^23,24^. This study found that the interval was not significantly different between the two groups.

One study suggests that poor compliance was an independent risk factor of inadequate bowel preparation, which has an approximate rate of 34.6%^25^. As the time interval increases, patients tend to forget the bowel preparation methods and precautions explained by the nurses, which eventually leads to a dramatic decline in their compliance. In this study, WeChat guidance could improve the compliance of the examinees. WeChat were used to communicate with the patients before the examination to inform them of the significance of colonoscopy. Furthermore, patients were reminded of the time of the examination, their diet and medication regimens in real-time. Additionally, they were assessed for their last bowel movement conditions.

There are some limitations to this study. First, WeChat guidance requires mobile phones and internet platforms to achieve mutual communication. However, some patients may fail to use the WeChat function due to the limitations of internet accessibility, internet traffic, and mobile phone models. Furthermore, WeChat counseling takes a lot of time from the nurses, which adds to their workload. These constraints need to be addressed by a more advanced contemporary media communication platform. Second, this is a single-center study, a multi-centered randomized control study is required to validate the results.

In conclusion, this study found that WeChat guidance might improve the quality of bowel cleansing, reduces adverse effects and increases the detection of colorectal adenoma. WeChat counseling might be helpful in colonoscopy and other clinical practice.

## Data Availability

All data generated or analysed during this study are included in this published article.
